# Microstructure and Tensile Properties of AZ61 Alloy Sheets Processed by High-Ratio Extrusion with Subsequent Direct Aging Treatment

**DOI:** 10.3390/ma11060895

**Published:** 2018-05-26

**Authors:** Cheng-Cheng Zhang, Hui-Yuan Wang, Min Zha, Cheng Wang, Jie-Hua Li, Zhi-Zheng Yang, Qi-Chuan Jiang

**Affiliations:** 1 Key Laboratory of Automobile Materials of Ministry of Education & School of Materials Science and Engineering, Nanling Campus, Jilin University, No. 5988 Renmin Street, Changchun 130025, China; cczhang15@mails.jlu.edu.cn (C.-C.Z.); wanghuiyuan@jlu.edu.cn (H.-Y.W.); minzha@jlu.edu.cn (M.Z.); Jiehua.Li@unileoben.ac.at (J.-H.L.); yangzz@jlu.edu.cn (Z.-Z.Y.); jqc@jlu.edu.cn (Q.-C.J.); 2 International Center of Future Science, Jilin University, Changchun 130012, China; 3 Institute of Casting Research, University of Leoben, 8700 Leoben, Austria

**Keywords:** magnesium alloy, high-ratio extrusion, aging, microstructure, tensile properties

## Abstract

A high extrusion ratio of 166:1 was applied to commercial AZ61 alloy in one step with an extrusion speed of 2.1 m·min^−1^. The effects of DA (direct aging) treatment on the microstructure and tensile properties of extruded alloy were investigated. The extruded alloy exhibits fine DRXed grains and the average grain size is ~11 μm. After DA treatment at 170 °C, the tensile strength and 0.2% offset yield strength is enhanced from 314 to 336 MPa and from 169 to 191 MPa respectively, sacrificing elongation from 26.5% to 23.3%. The grain size and texture distribution of extruded AZ61 scarcely evolve during the post aging treatment. However, the enhanced strength in peak-aged alloy is mainly caused by the high-density elliptical Mg_17_Al_12_ precipitates distributing uniformly along the grain boundaries or within the grains, by precipitation and dispersion hardening. Furthermore, the nano-sized precipitates effectively inhibit grains from coarsening by triggering pinning effects along the grain boundaries at elevated temperature. As a result, the peak-aged alloy exhibits a better superplasticity of 306.5% compared with that of 231.8% of extruded sample. This work provides a practical one-step method for mass-producing Mg alloy sheets with excellent tensile strength and ductility compared with those fabricated by conventional extrusion methods.

## 1. Introduction

There have been numerous efforts to process fine-grained or nano-structured Mg alloys, with the aim of exhibiting specific mechanical properties, such as high strength and ductility. Among various approaches in grain refinement, severe plastic deformation (SPD) methods have attracted considerable research interest. These SPD methods include high-pressure torsion (HPT) [1], equal channel angular extrusion (ECAE) or equal channel angular pressing (ECAP) [2,3], multiaxial forging (MAF) or multi-directional forging (MDF) [4,5], accumulative roll bonding (ARB) [6,7], as well as cyclic extrusion and compression (CEC) [8], etc. However, the application of SPD methods to mass production remains challenging at present. Hence, it is necessary to provide a simple and efficient processing method suitable for industry application.

Recently, the one-step high-ratio extrusion has received attention due to its flexibility for use on Mg alloys with a great diversity of shapes, such as sheets, rods, tubes, etc. It has been confirmed that an intense deformation using one pass is more effective in obtaining ultrafine grains and high fraction of high angle grain boundaries than an accumulated high strain with multiple passes [9,10]. Studies on high-ratio extrusion have been mainly concentrated on the AZ31 [11,12], ZK60 [13,14], AZ61 [15], AZ80 [15] and AZ91 [16,17] alloys. As reported by Chen et al. [11], the casting AZ31 ingots were first extruded or compressed with a small strain to obtain the conventional extruded billet and subsequently extruded in a one-step method, with the extrusion ratio from 7:1 to 100:1. A gradual grain refinement from ~25 to ~4 μm was achieved with the extrusion ratio increasing, when the extrusion temperature was 250 °C. Murai et al. [12] investigated the influence of extrusion ratio on the tensile properties of the extruded AZ31B alloy, where the extrusion ratio was set at 10:1, 50:1 and 100:1, respectively. Their results indicated that the influence of extrusion ratio on elongation was more significant than on tensile strength, and elongation tended to improve with an increase in extrusion ratio. Uematsu et al. [15] employed a high extrusion ratio of 133:1 on the AZ61 and AZ80 alloys with the working temperature controlled from 340 to 377 °C. Hereinto, significant grain refinement was achieved, with the average grain size of ~3.9 and ~4.3 μm for the AZ61 and AZ80 alloys, respectively. In addition, Watanabe et al. [17] confirmed that fine grained AZ91 alloy with the average grain size of ~1.7 μm could be achieved by hot extrusion alone at 250 °C in one step with an extrusion ratio of 44:1. A large elongation of over 300% at the high strain rate of 1 × 10^−2^ s^−1^ was obtained at around 275 °C in their work.

Nevertheless, studies on the influence of aging treatment have scarcely been concentrated on the AZ61 alloy processed by high-ratio extrusion. Moreover, most of the reported work on high-ratio extrusion mainly focuses on the superplasticity of extruded alloys. Therefore, the extrusion temperature is usually relatively low, varying from 250 °C to 350 °C to create significant grain refinement. This distinctly results in a very slow extrusion speed, e.g., the extrusion speed at 250 °C is only 20 mm·min^−1^ when the extrusion ratio is 100:1 [11]. Moreover, there is also an adverse effect that the product size is limited by the requirement of both high extrusion ratio and low extrusion temperature. This severely inhibits the application of one-step high-ratio extrusion for industrial production. Accordingly, it is necessary to provide a more practical method that can not only meet mass production, but also has excellent mechanical properties. Furthermore, it has been reported by Uematsu et al. [18] that precipitation takes place more significantly in direct aging than in solution and aging condition and further achieves a higher hardness and tensile strength.

Therefore, the present work primarily focuses on the microstructure and tensile properties of high-ratio extruded AZ61 alloy that followed by direct aging (DA) treatment, where a high-ratio extrusion of 166:1 was carried out in one step at a high extrusion temperature as well as fast extrusion speed.

## 2. Experimental Procedure

**Figure 1:**
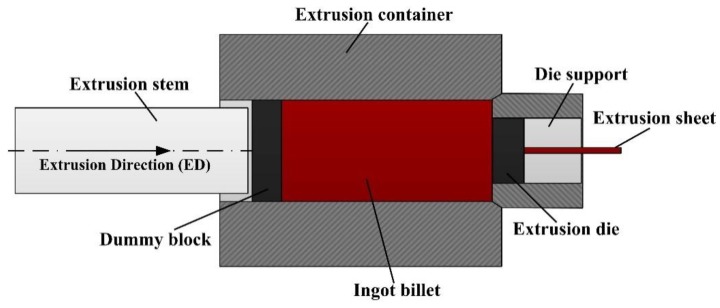
Sketch of the one-step extrusion process for AZ61 alloy.

The as-cast Mg-6.6Al-0.8Zn-0.3Mn (AZ61) alloy billets with a diameter of 95 mm were used in this study. After homogenization at 300 °C for 2 h and 400 °C for 5 h, the billets were extruded in a one-step process at an extrusion ratio of 166:1 to obtain a final sheet with the size of 0.95 mm in thickness and 45 mm in width, respectively. For better understanding the process, a sketch of the one-step extrusion process is illustrated in Figure 1. The homogenized alloy was extruded at 390 °C and the extrusion flow rate was set at 2.1 m·min^−1^. The actual temperature of the deformation zone in the extrusion process was measured using a thermocouple, which was installed inside the die. It turned out that the temperature increased to ~420 °C because of plastic deformation and friction in the extrusion process.

After processing, the extruded sheet was directly aged at 170 °C in the air furnace, as followed by naturally air cooling. The aging response was characterized in the mechanically polished samples using Vickers microhardness testing (1600-5122 VD MICROMET 5104, New York, NY, USA) at a load of 50 g for 15 s. Tensile specimens of the extruded and peak-aged samples were tested along the extrusion direction (ED) on INSTRON 5869 with a strain rate of 1.0 × 10^−3^ s^−1^ at room temperature and 300 °C, respectively. The gauge size of both cases is the same with 30 × 10 × 0.95 mm^3^. The microstructure was observed by an optical microscope (OM, Carl Zeiss Axio Imager A2m, Göttingen, Germany) and a scanning electron microscope (SEM, VEGA3 XMU, TESCAN, Brno, Czech). The phase constituents were analyzed on the X-ray diffraction (XRD, D/Max 2500PC, Rigaku, Tokyo, Japan) using Cu Kα radiation. The scanning electron microscope (SEM, VEGA3 XMU, TESCAN, Czech) equipped with an Oxford Instruments NordlysNano EBSD detector using Channel 5.0 data acquisition software was used to observe the grain structure and microtexture evolution. The accelerating voltage and step size was 20 kV and 0.5 μm, respectively. Nano Measurer 1.2 was used to obtain the average grain size and distribution of the Mg matrix and precipitates [19], which was derived from at least eight micrographs.

## 3. Results and Discussion

**Figure 2:**
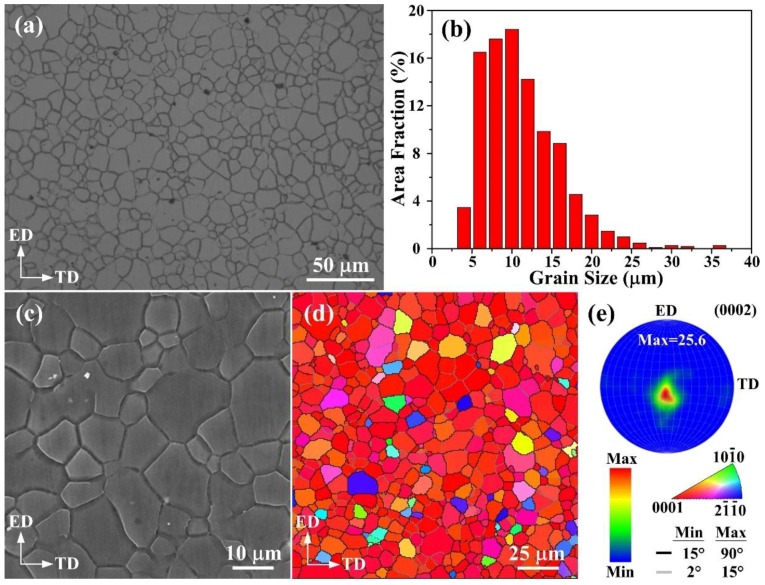
(**a**) Optical image, (**b**) grain size distribution, (**c**) SEM micrograph, (**d**) EBSD IPF map and (**e**) corresponding (0002) pole figure of the extruded AZ61 alloy.

Figure 2a shows the optical micrograph of the extruded sample. Fine and equiaxed grains distribute uniformly within the whole area, which indicates that dynamic recrystallization (DRX) takes place completely during the extrusion process. It is noteworthy that the grain size of extruded AZ61 alloy has a narrow range mainly from 5 to 15 μm, revealing an average grain size of ~11.1 μm (Figure 2b). Chung et al. [20] reported that the mean grain size of the 3-pass and 4-pass ECAPed AZ61 alloy was ~11.2 and ~10.6 μm, respectively. Consequently, the grain size of this work is comparable to their values and much finer than those fabricated by conventional extrusion process (20–35 μm) [21,22,23]. Furthermore, it is widely accepted that high strength and high ductility at room temperature is generally achieved by grain refinement [24]. Accordingly, the relatively finer grains in this work are conducive to a better combination of tensile strength and ductility. Generally, the relationship between true strain and extrusion ratio can be expressed as follows:

*ε* = *lnR_e_* (1)

where *ε* is the true strain and *R_e_* is the extrusion ratio [11]. Hence, the present work obtains a true strain of about 5.1. Lin et al. [16] described a similar work that the AZ91 alloy was extruded with a reduction ratio of 166:1 at 250–350 °C. In their work, a rod billet with a diameter of 65 mm was extruded into a plate with width of 10 mm and thickness of 2 mm. Note that their extrusion size is much thicker and smaller that of 0.95 mm × 45 mm in this work. This confirms the practicability of fabricating Mg alloys sheets in thickness of less than 1 mm by a one-step extrusion process.

Figure 2c exhibits the typical SEM feature of the extruded AZ61 alloy. It is evident that few precipitates can be seen in the Mg matrix, which implies that most of Mg_17_Al_12_ phases have dissolved into the α-Mg matrix. Figure 2d,e illustrates the EBSD inverse pole figure (IPF) map and corresponding (0002) pole figure obtained from extrusion direction (ED)-transverse direction (TD) plane of extruded AZ61 alloy. High-angle grain boundaries with misorientations between 15° and 90° are colored in black, while low-angle grain boundaries are marked in gray with misorientations in the range from 2° to 15°. It demonstrates that the extruded specimen has a texture at the centre of the (0002) pole figure with little spread, in which the peak intensity is 25.6, indicating a basal texture. This is different from the conventional extruded texture where the basal planes parallel to the ED with a spread toward the TD. Similarly, a strong basal texture with a roughly circle-shaped distribution of {0001} orientation has also been reported in the hot-extruded AZ61 alloy [25].

**Figure 3:**
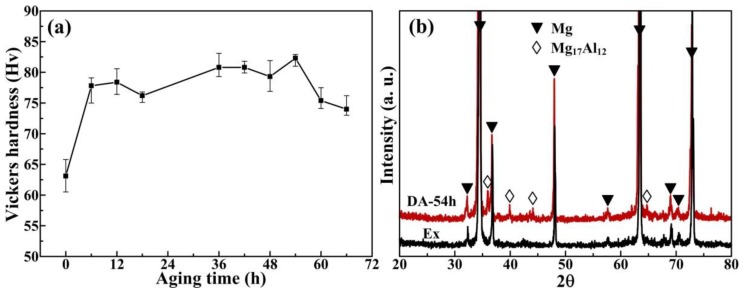
(**a**) Vickers hardness with aging time at 170 °C and (**b**) XRD patterns of the extruded and DA-54h AZ61 alloy.

To further improve the mechanical properties of the extruded AZ61 alloy, direct aging (DA) treatment was carried out at 170 °C in the present work. The change in Vickers hardness with the aging time is shown in Figure 3a. It shows that an obvious age-hardening is obtained where the micro-hardness increases from 63.1 (aged for 0 h) to the peak value of 82.3 at 54 h (DA-54h). Figure 3b shows the XRD analysis of the extruded and DA-54h samples. The typical peaks of Mg_17_Al_12_ phases in the DA-54h sample (red line) are more obvious in comparison with the extruded sample (black line). Hence, the increased micro-hardness is mainly attributed to the effective precipitation of Mg_17_Al_12_ particles by DA treatment. In addition, there are no peaks containing Zn in both cases, originating from the high solubility of Zn in Mg and relatively low content in this work, so most of Zn has dissolved into the matrix.

**Figure 4:**
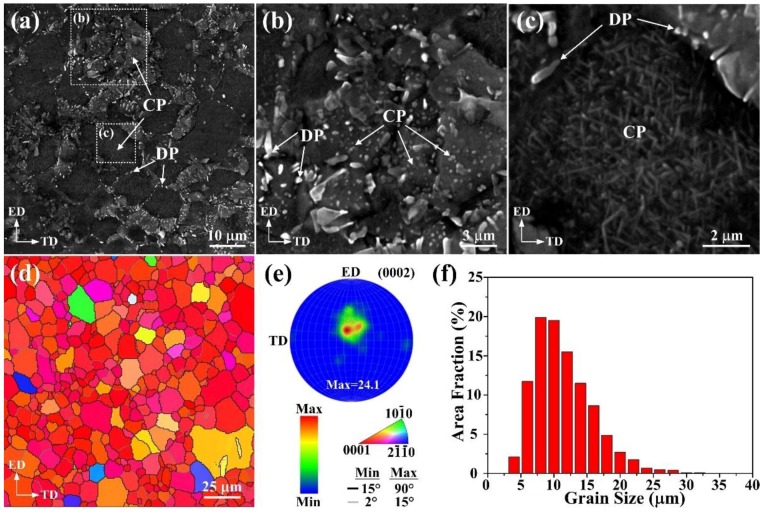
(**a**) Low magnification, (**b**,**c**) high magnification SEM micrographs (CP: continuous precipitation, DP: discontinuous precipitation), (**d**) EBSD IPF map, (**e**) (0002) pole figure and (**f**) grain size distribution of DA-54h.

Figure 4a shows the SEM micrograph of the DA-54h specimen. Plenty of second phases have precipitated after DA treatment. Two types of precipitation morphology can be observed coexistent in the matrix, i.e., discontinuous precipitation along the boundaries and continuous precipitation within the grains. To further observe the two different types of precipitates clearly, high magnified SEM images are given in Figure 4b,c. It can be seen that most of the discontinuous precipitates along the boundaries have an elliptical morphology with the size ranging from 0.2 to 1.5 μm and the average value is ~0.5 μm (Figure 4b). This result is different from the coarse lamellar morphology of discontinuous β-Mg_17_Al_12_ phases that the precipitates generally grow from the grain boundaries into the grain interiors. The elliptical morphology in this work implies that the growth process of discontinuous precipitation is effectively inhibited. It is recognized that vacancies, dislocations and twins induced by deformation can act as the precipitation nuclei during the post heat treatment [26]. Although most of the deformation twins have been consumed by the DRX (Figure 2a,c) process, it is believed that there still exists a high density of vacancies and dislocations in the extruded condition, as the extrusion ratio is as high as 166:1. Furthermore, the distribution of solute atoms in the matrix is generally more uniform with the introduction of high-ratio extrusion compared with conventional extrusion. Hence, large amounts of continuous Mg_17_Al_12_ precipitates can be observed to occupy the remaining areas within the grains when direct aging treatment is performed on the extruded sample. The results demonstrate two distinct morphologies of continuous precipitates in the present work. One is the elliptical morphology with the diameter less than 1 μm (Figure 4b), which is similar to the discontinuous precipitation. The other exhibits a Widmanstätten structure (Figure 4c), which is the dominant morphology for continuous precipitates in the aging temperatures between 150 and 250 °C [27]. In addition, the uniform precipitation with small size is also attributed to the relatively low temperature of aging treatment, which is 170 °C in this work.

Figure 4d,e exhibits the EBSD analysis results of the DA-54h sample. Note that there is no significant evolution in the peak intensity, implying that the texture of extruded alloy scarcely evolved during the post heat treatment of DA. Additionally, the grain size distribution of the DA-54h sample is given in Figure 4f. It demonstrates that the grain size mainly distributes in a range from 5 to 15 μm with an average value of ~11.5 μm. Therefore, it is concluded that apart from the precipitation of Mg_17_Al_12_ phases, the DA-54h sample has a similar texture and grain size distribution to the extruded AZ61 alloy.

**Figure 5:**
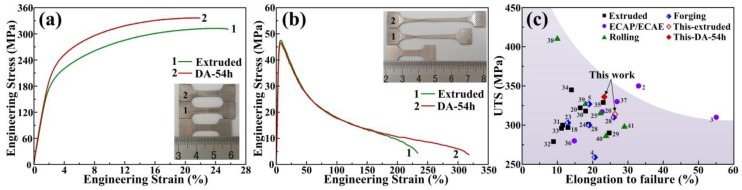
(**a**) Tensile engineering stress-strain curves of the extruded and DA-54h alloy at room temperature and (**b**) at 300 °C (insets: comparison of specimens before and after deformation), (**c**) ultimate tensile strength (UTS) as a function of elongation to failure of AZ61 alloy processed by various methods in the literature for comparison with this work.

In order to study the effect of DA treatment on the mechanical properties, tensile tests of the extruded and DA-54h samples were carried out at room temperature and 300 °C, respectively. The results of typical tensile engineering stress-strain curves are shown in Figure 5a,b with corresponding tensile properties listed in Table 1. It is evident that the extruded sample shows a good combination of strength and ductility, with the tensile strength σ_b_ of 314 MPa and the elongation to failure ε_f_ of 26.5%. After subsequent heat treatment of DA at 170 °C for 54h, the tensile strength of DA-54h further enhances to 336 MPa. Jiang et al. [28] demonstrated a similar improvement of tensile strength by aging treatment for AZ61 alloy, which was increased from 310 to 327 MPa. However, the sacrifice of their elongation is obvious from 26% to 19%. It is noteworthy that the elongation of DA-54h is 23.3%, indicating a decrease of 3.2% compared with the extruded sample. Hence, the sacrifice of ductility in this work is smaller than that of 7% in their work. Compared with the coarse lamellar discontinuous precipitation in their work, the precipitates in this work are much finer in size (~0.5 μm) and more uniform in distribution. Since smaller and more numerous precipitate particles are better able to resist dislocation slip, they are more conducive to the mechanical properties [27]. Also, the 0.2% offset yield strength *σ*_0.2_ improved from 169 to 191 MPa after DA treatment, mainly due to the precipitation hardening of Mg_17_Al_12_ phases, as there is no distinct difference in the texture and grain size distribution between both cases. In addition, the dispersive second phases of high volume fraction also contribute to the dispersion hardening by the Orowan bypass mechanism. For the tensile tests at 300 °C, the tensile strength of extruded sample is 47 MPa and the elongation to failure is 231.8% (Figure 5b and Table 1). An obvious improvement of elongation is achieved in DA-54h sample, which is 306.5% with a comparable tensile strength of 48 MPa.

Additionally, the tensile properties of AZ61 alloy processed by various methods such as conventional extrusion [18,20,23,29,30,31,32,33,34,35], ECAP [2,3,20,36,37], multi-pass rolling [25,38,39,40,41] and forging [4,5,28] in the literature, are given in Figure 5c for comparison with this work. Note that the excellent balance of tensile strength and ductility in this work shows significant advantage compared with the conventional extrusion and is even comparable to the ECAP deformation. Accordingly, the one-step process with high extrusion ratio followed by direct aging treatment is proven to be a simple, economical and highly effective method for producing Mg alloy sheets with excellent mechanical properties.

**Figure 6:**
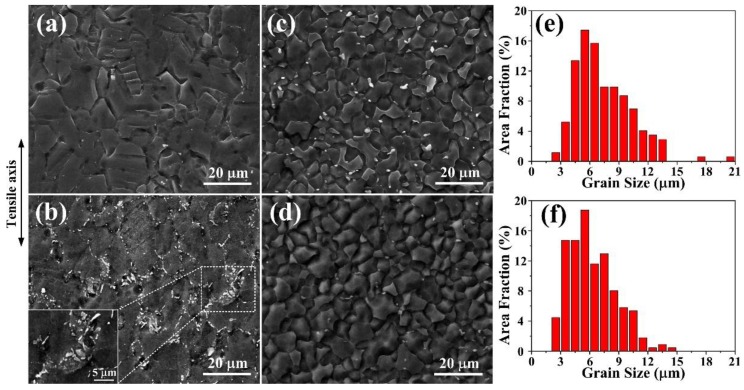
SEM micrographs of the fractured samples in the gauge section after tensile deformation of (**a**) extruded, (**b**) DA-54h at room temperature and (**c**) extruded, (**d**) DA-54h at 300 °C with corresponding grain size distribution in (**e**,**f**), respectively.

Figure 6a,b shows the fracture micrographs of the extruded and DA-54h samples in the gauge section after tensile deformation at room temperature. As the initiation of microcracks is strongly connected to the presence and morphology of second phases, the reduction in ductility in the DA-54h sample should be mainly attributed to the existence of coarse micron-sized Mg_17_Al_12_ phases (Figure 6b), which may result in dislocation pile-ups and hence increase stress concentration in the neighborhood, acting as potential sources of breakage during tensile deformation. Figure 6c,d demonstrates the fracture micrographs of the extruded and DA-54h samples after tensile deformation at 300 °C, and the corresponding grain size distribution are shown in Figure 6e,f, respectively. Note that fine equiaxed grains with the grain size varying from 3 to 10 μm can be seen in the extruded (Figure 6c,e) and DA-54h (Figure 6d,f) samples, indicating the occurrence of dynamic recrystallization in both cases. However, it should be noted that there is a more uniform and finer microstructure with an average size of ~6 μm in the DA-54h alloy, which is believed to be conducive to obtaining a better superplasticity. Moreover, it is evident that most of the Mg_17_Al_12_ phases of DA-54h have dissolved back into the matrix and re-precipitated on the grain boundaries after tensile deformation at 300 °C (Figure 6d). It is well known that the mobility of grain boundaries and dislocations can be restricted due to the pinning force originated from particles [42]. The uniform nano-sized Mg_17_Al_12_ particles of DA-54h play a significant pinning effect on the grain boundaries, effectively inhibiting grains from coarsening in the process of tensile deformation at 300 °C and resulting in a better superplasticity of 306.5% compared with the extruded AZ61 alloy (231.8%, Figure 5b and Table 1).

Overall, the one-step extrusion process with a high extrusion ratio can significantly improve both the strength and ductility of AZ61 alloy in this work. Both the tensile strength and yield strength are further enhanced after DA heat treatment, with minimally sacrificing elongation. Moreover, the precipitation of nano-sized Mg_17_Al_12_ phases with uniform distribution allows better superplasticity, by effectively inhibiting the grain growth. Therefore, there is enormous potential for the one-step high-ratio extrusion with subsequent heat treatment of DA for the industrial production of Mg alloy sheets with excellent balance of tensile strength and ductility.

## 4. Conclusions

A high-ratio extrusion of 166:1 was successfully performed on the AZ61 alloy in one step without cracks for obtaining an alloy sheet less than 1 mm with a mass-producing speed of 2.1 m·min^−1^. The extruded alloy exhibits fine DRXed grains and the average grain size is ~11 μm, resulting in an advantageous balance of strength and ductility. The tensile strength and 0.2% offset yield strength is further enhanced from 314 to 336 MPa and from 169 to 191 MPa respectively after DA treatment, with a little sacrifice of elongation from 26.5% to 23.3%. Moreover, the peak-aged alloy shows a better superplasticity of 306.5% compared with that of 231.8% of extruded sample in the tensile deformation at 300 °C. Since the grain size and texture distribution of extruded AZ61 scarcely evolved during the post-aging treatment, the enhanced strength in peak-aged alloy is mainly due to the high-density elliptical Mg_17_Al_12_ precipitates. The nano-sized precipitates distributing uniformly along the grain boundaries or in the grain interiors played a significant role in the precipitation hardening and dispersion hardening, and further inhibit grains from coarsening by triggering pinning effects along the grain boundaries at elevated temperatures.

## Figures and Tables

**Table 1:** Tensile properties of the extruded and DA-54h AZ61 alloy at room temperature and 300 °C.

Samples	Room Temperature			300 °C	
	Tensile Strength, σ_b_/MPa	Yield Strength, σ_0.2_/MPa	Elongation to Failure, ε_f_/%	Tensile Strength, σ_b_/MPa	Elongation to Failure, ε_f_/%
Extruded	314 ± 2	169 ± 6	26.5 ± 0.7	47 ± 1	231.8 ± 1.8
DA-54h	336 ± 1	191 ± 3	23.3 ± 1.3	48 ± 1	306.5 ± 11.5
